# Electron transfer properties of biobased and biodegradable polymers: PHA as a case study

**DOI:** 10.1007/s00894-026-06775-8

**Published:** 2026-05-25

**Authors:** Emiliano Perez-Sanchez, Alexis Caballero, Ana Martínez

**Affiliations:** Departamento de Materiales de Baja Dimensionalidad, Instituto de Investigaciones en Materiales, Universidad Nacional Autónoma de México, 04510 Mexico, México

**Keywords:** Toxicity, Oxidation, Chemical reactivity parameters, Polyhydroxyalkanoates, FEDAM

## Abstract

**Context:**

Plastics can remain unchanged for long periods of time, thus generating large amounts of waste. Due to mechanical or chemical processes, plastics can be broken down into micro- and nanoplastics. To analyze the oxidative stress that nanoplastics might produce, 21 oligomers of polyhydroxyalkanoates (PHA) are investigated. We used oligomers of PHA as models of nanoplastics. We determined the electron donor–acceptor capacity using the Full Electron Donor–Acceptor Map described previously. All the investigated PHA exhibit the same electron donor and acceptor properties, regardless of the system size. They are all the worst electron donors and acceptors of the systems under consideration. These results indicate that these nanoplastics are not capable of electron transfer and, therefore, it is not evident that they can contribute to oxidative stress in animals and plants. The interaction of PHA with phloretin was also analyzed, since PHA is functionalized with phloretin to obtain packaging materials. The electron transfer capacity of PHA is enhanced in the presence of phloretin. This research suggests that PHA nanoplastics do not produce oxidative stress, which is important for further studies that are still required.

**Methods:**

All DFT computations were performed using the Gaussian16 at def2-TZVP/ω-B97XD level of theory without symmetry constraints.

## Introduction

Plastics are everywhere and they represent serious environmental concern. The production is massive and, once no longer in use, the poor disposal of these materials causes them to become persistent in ecosystems [1–5]. Plastics are polymers, which are very stable materials and can remain unchanged for long periods of time [6, 7]. Conventional petrochemical-based plastics degrade slowly, and so they accumulate in terrestrial and aquatic environments, causing widespread pollution [8–11]. Plastic pollutants disrupt wildlife because they can become entangled, thus causing physical damage.

Due to mechanical or chemical processes, plastics can break down into smaller fragments, forming micro and nanoplastics. Micro- and nanoplastics are difficult to detect (especially nanoplastics), nevertheless, samples have been identified in almost all natural and human environments [12–15]. Previous studies indicate that nanoplastics can affect animals, but there are gaps in our knowledge on their adverse health effects [16–21]. Apparently, their presence in the bodies of animals can provoke inflammation, which then leads to oxidative stress, but there are doubts over whether they can cause adverse chemical reactions that can be detrimental to human, animal, or plant life [22–27]. Preceding reports indicate that chemical reactivity is regarded as the first condition that must be met for a substance to be considered toxic [28, 29]. Chemical reactions produce toxicity when they involve biomolecules. Previous investigations have considered this idea for studying the potential toxicity of nanoplastics, and also analyze their capacity to produce oxidative stress through electron transfer reactions [28, 30]. Although these results indicate that there are no chemical reactions with nanoplastics, this must be demonstrated experimentally.

In spite of all the studies reported to date, there are no theoretical investigations considering polyhydroxyalkanoates (PHA), which are important biodegradable plastics [31, 32]. Biodegradable plastics have emerged as a sustainable alternative to non-biodegradable polymers [32, 33]. With these plastics, environmental damage is reduced. Biodegradable polymers can be derived from fossil or natural sources, and in some cases from both, as is the case with PHA [32, 34]. It should be noted that the fact that a polymer is obtained from natural sources (biopolymers) does not guarantee that it will also be biodegradable [35]. The biodegradable nature of a polymer depends primarily on its chemical composition [36], such as the presence of hydrolysable linkages like ester, ether, or amide bonds. This is independent of whether the polymer is of natural or synthetic origin [37].

**Scheme 1:**
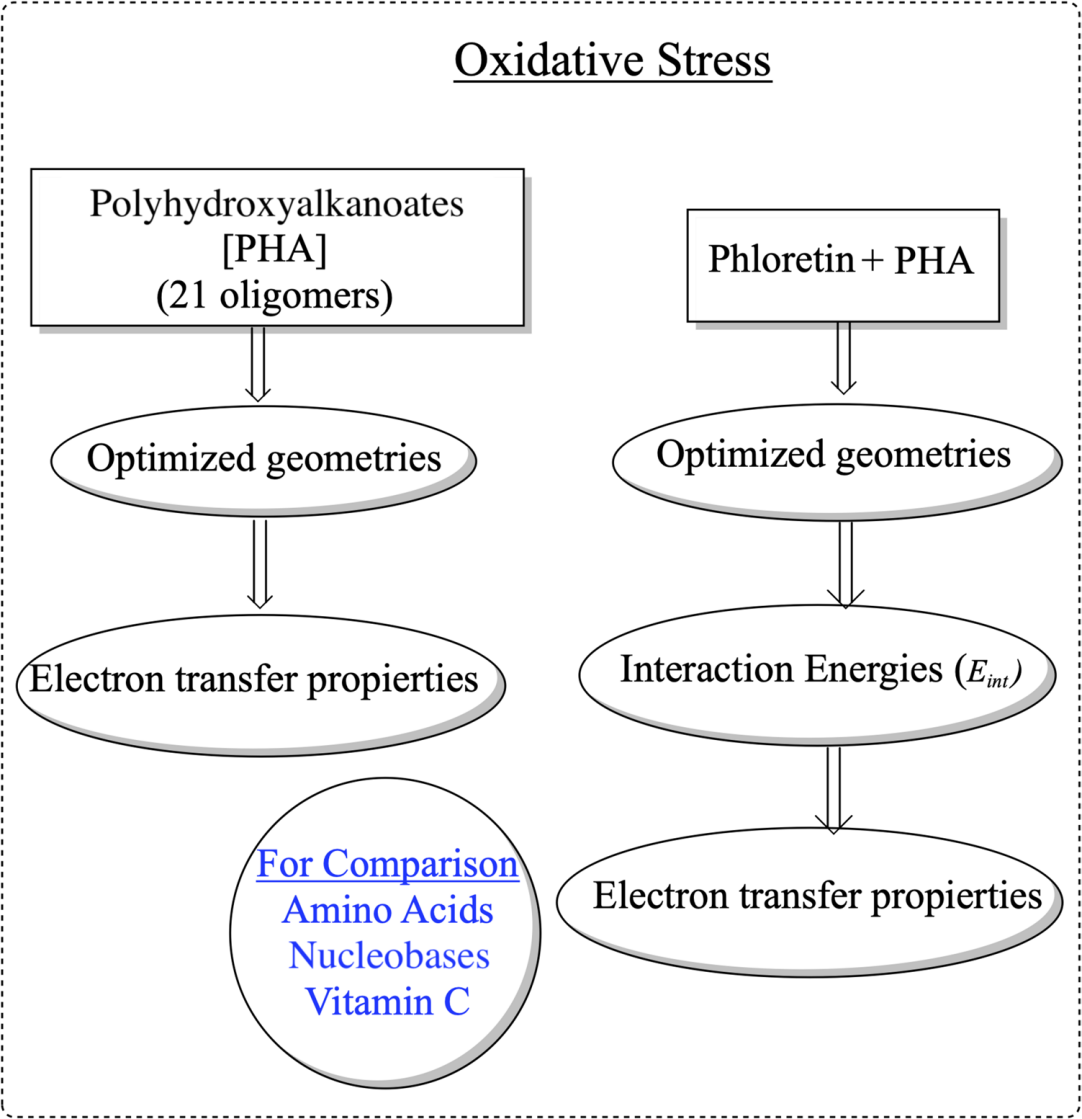
Schematic representation of the research objective and the sequence of steps

It is important to understand the chemical reactivity of nanoplastics coming from bioplastics, as their formation is in many instances a prerequisite for mineralization [38]. In this investigation, we analyzed the antioxidant capacity of nanoplastics of PHA, using oligomers as models (PHA). We investigate the electron transfer as the antioxidant mechanism of 21 oligomers and four selected copolymers of PHA. We also analyzed PHA functionalized with phloretin since it was previously reported that this combined system can possess antioxidant properties [39]. Scheme 1 presents the steps undertaken in this work.

The results reported here indicate that these nanoplastics are not capable of transferring electrons, and therefore, it is not obvious that they can contribute to oxidative stress in animals and plants.

## Computational details

**Fig. 1:**
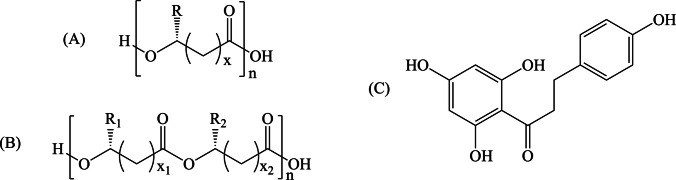
General molecular formula of (**A**) PHA, copolymers of PHA (**B**) and phloretin (**C**). Typically, x = 1–8 but we used systems with x, x_1_ and x_2_ equal to 1

Density Functional Theory calculations with the Gaussian16 code [40] were performed at a def2-TZVP/ω-B97XD level of theory [41, 42]. This functional accounts for dispersion interactions. Since PHA undergoes degradation and microplastic formation in aqueous solutions, water was considered as a solvent to perform all calculations. Solvent effects were included during the optimization and in all calculations using the integral equation formalism of polarizable continuum model (IEF-PCM) [43–46]. Unrestricted calculations were performed and local minima were identified by the number of imaginary frequencies (NIMAG = 0). For the interaction with phloretin, several initial conformations were considered, with different approximations to oligomers forming different bonds, and only the most stable structures are hereby presented. Oligomers have been used successfully to analyze various nanoplastics properties [28–31, 47]. The general structure of PHA and copolymers of PHA that we used are reported in Fig. 1. Phloretin’s structure is also reported in Fig. 1.

In order to model nanoplastics, we use PHA ranging from four (monomer) up to ten carbon atoms in the main chain. For R, we use from methyl to heptyl groups (see Table 1).

**Table 1:** PHA oligomers under study


Label	R group	Carbon no	PHA polymer
PHA-methyl	Methyl	C_4_	Poly(3-hydroxybutyrate)
PHA-ethyl	Ethyl	C_5_	Poly(3-hydroxyvalerate)
PHA-propyl	Propyl	C_6_	Poly(3-hydroxyhexanoate)
PHA-butyl	Butyl	C_7_	Poly(3-hydroxyheptanoate)
PHA-pentyl	Pentyl	C_8_	Poly(3-hydroxyoctanate)
PHA-hexyl	Hexyl	C_9_	Poly(3-hydroxynonanoate)
PHA-heptyl	Heptyl	C_10_	Poly(3-hydroxydecanoate)

To analyze the electron transfer reactions, vertical ionization energy (IE) and vertical electron affinity (EA) are calculated as follows:

1 $$Y\rightarrow Y^{1+} + 1e^{-} IE = E (Y^{1+}) - E (Y)$$

2 $$Y^{1-} \rightarrow Y + 1e^{-} EA = E (Y) - E (Y^{1-})$$

**Fig. 2:**
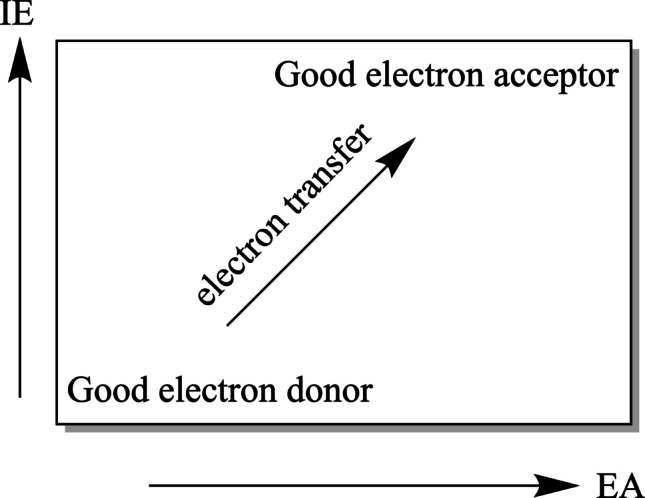
Full Electron Donor–Acceptor Map (FEDAM)

With these values, we obtain the Full Electron Donor Acceptor Map (FEDAM) as shown in Fig. 2. EA values are plotted in the X-axis, and IE values are plotted in the Y-axis. FEDAM was previously defined, and it is a powerful tool to compare the capacity to donate or accept electrons of different systems [48]. Good electron acceptors will have large EA values, and good electron donors will present small IE values. We can classify systems using this map, since all molecules with large EA are good electron acceptors (they are on the right side of the map) and those with small IE are good electron donors (in the lower section of the map). Electrons will be transferred from good electron donors to good electron acceptors, as indicated with the arrow in Fig. 2.

Interaction energies (*E*_*int*_) between phloretin and PHA oligomers were obtained as follows:

3 $$E_{int} = [E(PHA-R) + E(phloretin)] - E(PHA-R- phloretin)$$

The equation corresponds to the sum of the energies of reactants (PHA-R and phloretin) minus the energy of the product (PHA-R-phloretin). Positive values indicate that product (PHA-R- phloretin) is more stable than reactants.

## Results and discussion

**Fig. 3:**
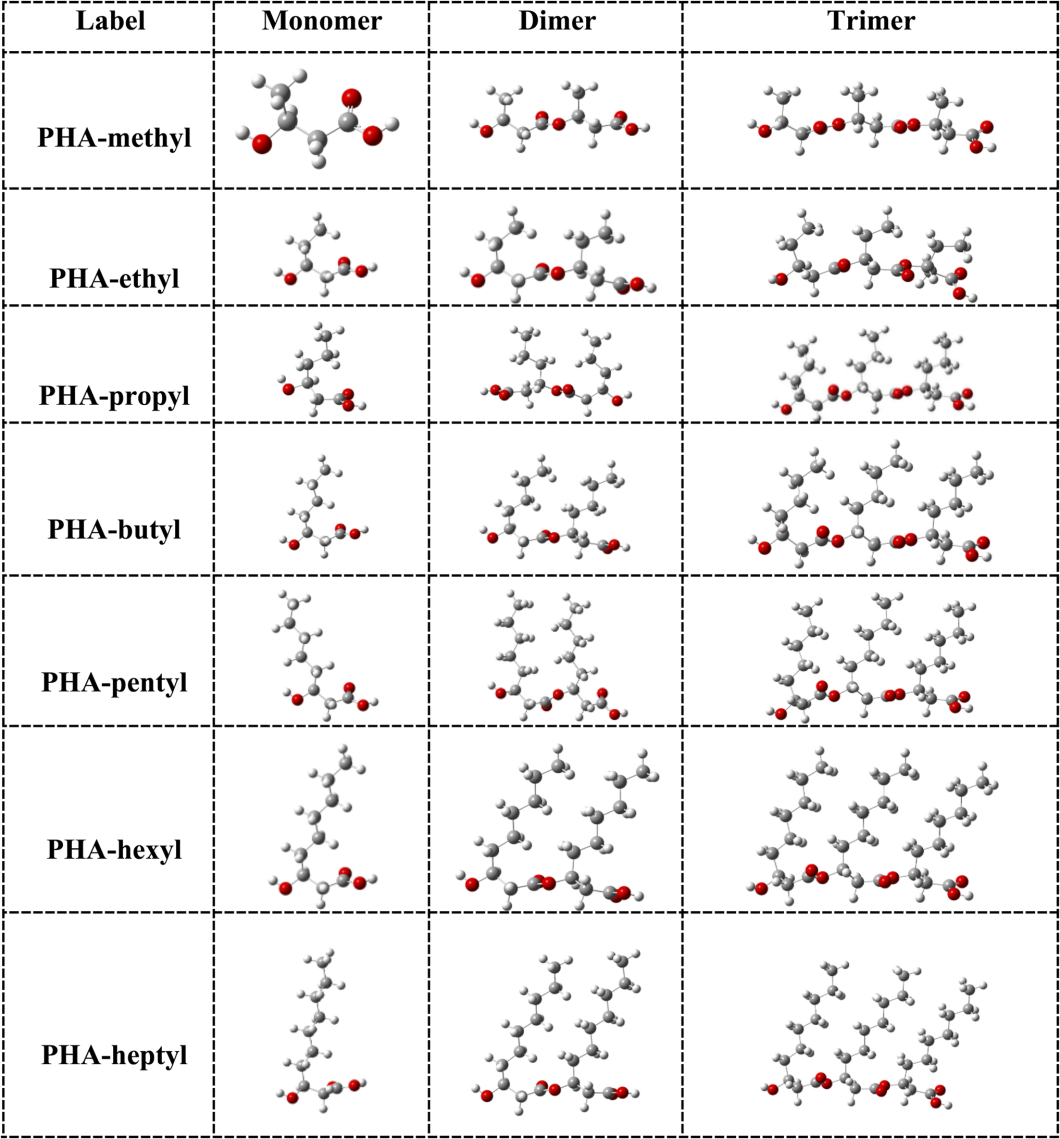
Optimized structures of PHA. Grey spheres represent carbon atoms, white and red spheres represent hydrogen atoms and oxygen atoms, respectively

**Fig. 4:**
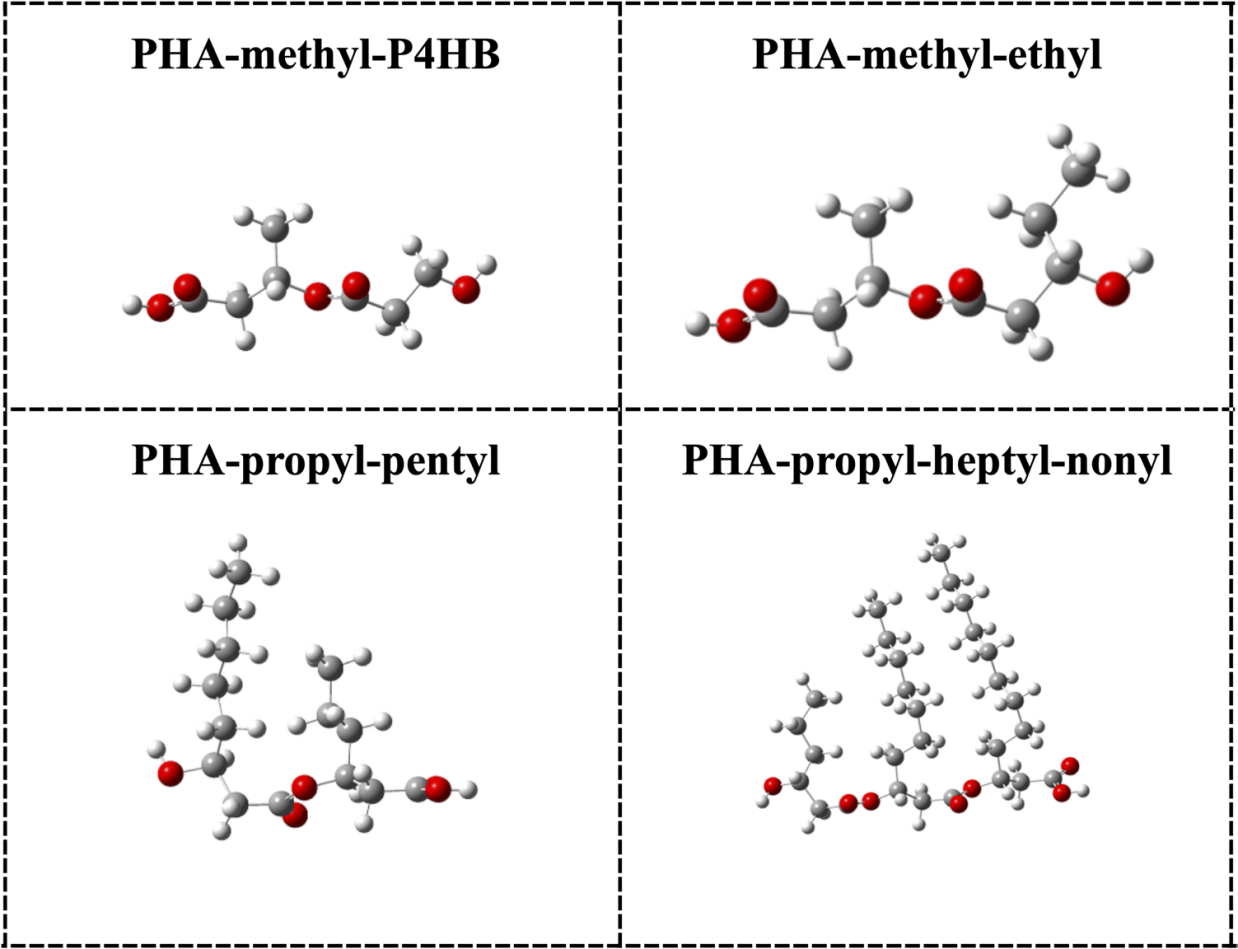
Optimized structures of PHA that represent co-polymers (the monomer). Grey spheres represent carbon atoms, white and red spheres represent hydrogen atoms and oxygen atoms, respectively

Optimized structures of all the oligomers under study are reported in Figs. 3 and 4. In these structures, R group chains are parallel to each other. We obtained other optimized structures with the R group chains in different positions (not shown), but they were found to be less stable. The main chain in all systems is quasi linear. The C–C and C-O bond distances are 1.5 and 1.2 Å, respectively. The idea is to analyze the chemical properties of PHA with respect to the size of the system. There are no C–C double bonds in these structures, and so little reactivity is expected. The most reactive part of the molecules would be the oxygen atoms, but they are forming hydroxyl and carbonyl groups and so they are expected to be stable.

The co-polymers contain two or more different monomers. They are arranged randomly in block patterns. Since the first study that reports the productions of copolymers from microorganisms, very few investigations of these systems have been reported [49]. In Fig. 4, we report four co-polymers that we investigated. The main carbon chain is also linear, and the bond distances are similar to those of Fig. 3. These optimized structures correspond to oligomers representing the monomer of the co-polymer.

**Fig. 5:**
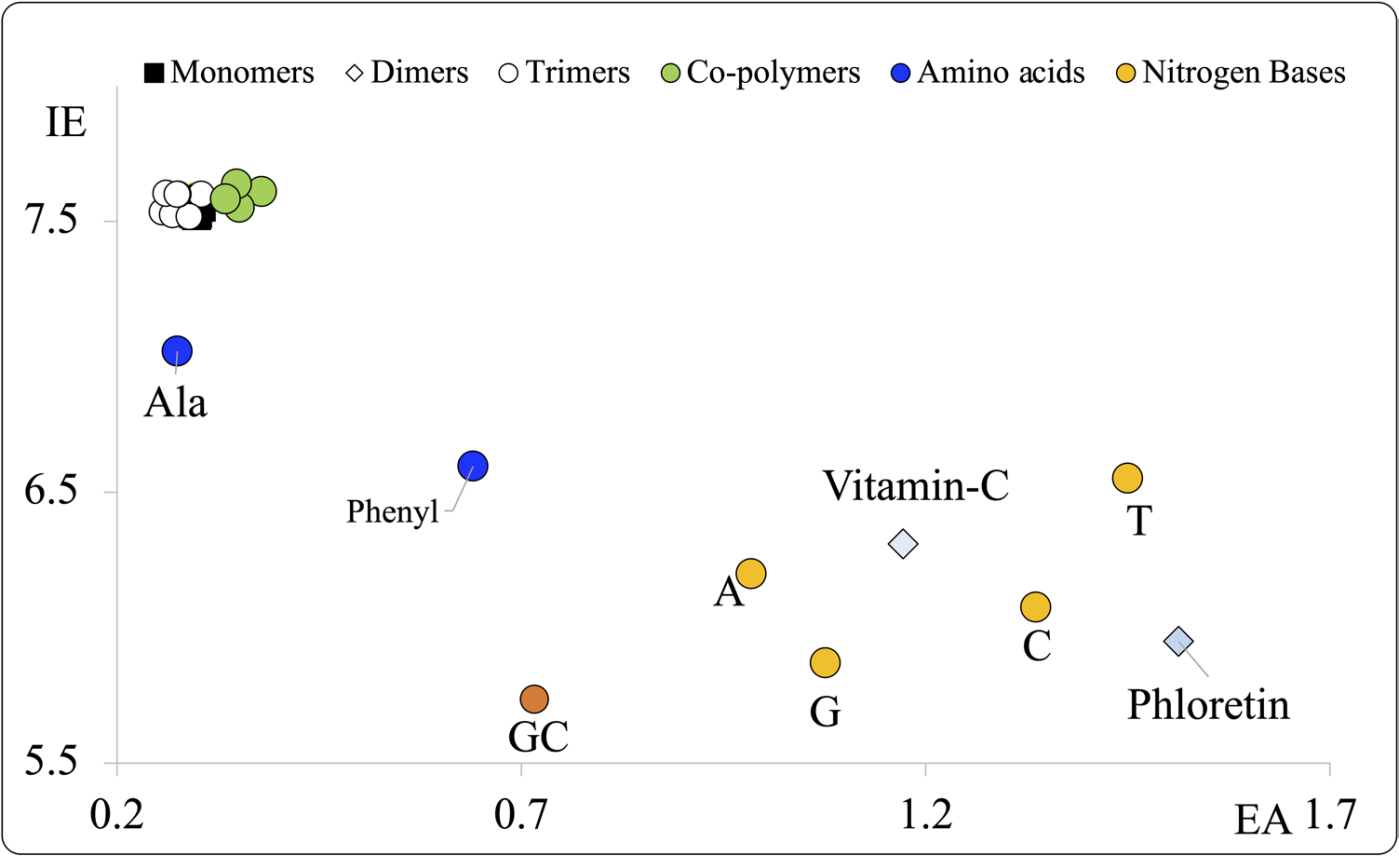
FEDAM of the most stable optimized structures of oligomers under study, as well as other molecules such as amino acids, nitrogenous bases, vitamin C and phloretin. GC is the guanine-cytosine base pair. A is adenine, G is guanine, C is cytosine, T is thymine, Ala is alanine and Phenyl is phenylalanine. All values are in eV. IE and EA are calculated with Eqs. (1) and (2)

The chemical reactivity parameter that we analyze for these systems is the electron donor acceptor capacity, that is related to the oxidative stress. To do this, we used the FEDAM as explained previously. In Fig. 5, we report the FEDAM of all the systems under study. It is important to compare the electron transfer capacity of the systems studied with that of other molecules. For this purpose, we used vitamin C and phloretin, previously reported as antioxidants. This comparison allows us to determine if our systems could also be effective antioxidants. Biomolecules such as two amino acids, four nitrogenous bases, and guanine-cytosine allow us to investigate whether the systems studied could transfer electrons to these biomolecules, as this could indicate a possible process of oxidation.

The first thing to note is that all PHA (oligomers of polymers and copolymers) are found in the same section of the FEDAM. They all exhibit the same electron donor and acceptor properties, regardless of the system size. They are all the worst electron donors (highest EI values) and the worst electron acceptors (lowest EA values). This means that these systems are not able to transfer electrons, and so they will not donate or accept electrons from other systems such as the nitrogenous bases or the amino acids. The PHA are quasi-inert according to the FEDAM compared to the other molecules that are also included. The first conclusion we have is that these nanoplastics are unlikely to induce oxidative stress through the charge transfer mechanism.

Comparing with previous results, electron transfer reactions have been reported for other oligomers [29] and they were also found to be not good electron transfer systems. So far, quantum chemical calculations from previous reports have indicated that oligomers do not contribute to the oxidative stress, since the electron transfer is not spontaneous. Polyethylene terephthalate (PET) oligomers are an exception, as they were found to be good electron acceptors and might cause oxidative stress by oxidizing biomolecules [30]. The electron transfer capacity of PLA oligomers has been also analyzed, and it was determined to be a better electron acceptor than other oligomers, in particular polystyrene [29].

One difference between the results reported previously and those presented here for PHA is the dependence of the electron transfer properties on the size of the systems. For oligomers of polyethylene, the ability to donate and also the ability to accept electrons decreases as the size of the system increases [30]. As a result, they become more reductant molecules and are therefore unlikely to oxidize other systems. For PET’s oligomers the results are similar, and so the ability to accept or donate electrons is related with the size of the oligomer. This is not the case for PHA since for these systems, the size doesn’t matter.

**Fig. 6:**
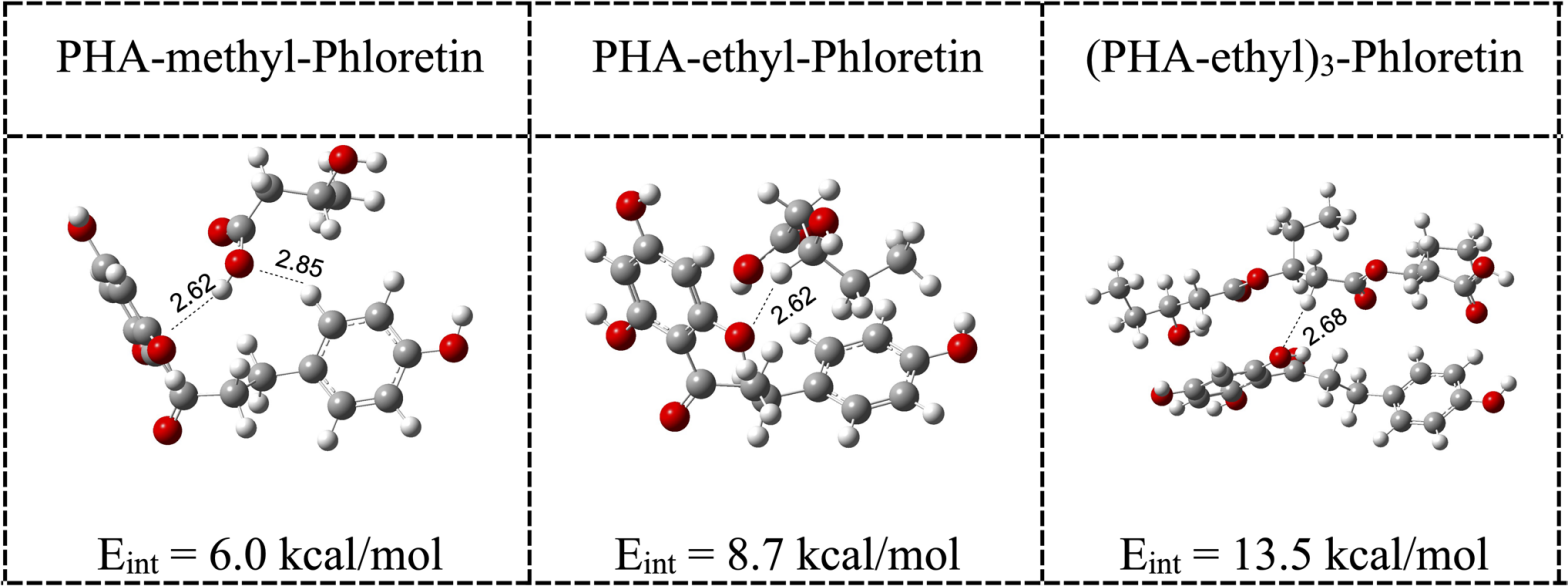
Optimized structures of oligomers interacting with phloretin. Grey spheres represent carbon atoms, white and red spheres represent hydrogen atoms and oxygen atoms, respectively. Interaction energies *(Eint*) calculated according to Eq. (3) are reported in kcal/mol

As can be seen in Fig. 5, phloretin is a good electron acceptor, with one of the highest EA values, and a good electron donor, with one of the lowest EI values. This could explain previous results that reported phloretin as an antioxidant that helps prevent oxidative stress [50]. It can accept or donate electrons from other molecules, oxidizing or reducing them. Returning to PHA, we know that they are generated by microorganisms. They are biodegradable and biocompatible biopolymers with diverse and versatile properties. They constitute an important component in the design of novel nanomaterials and have also been used in the formulation of environmentally friendly, bio-based biodegradable products, which serve as an alternative in products such as packaging, as well as in medical applications [51–55]. It was reported that PHA functionalized with phloretin, a good antioxidant molecule, can act against food-borne pathogen bacteria, thus preventing food spoilage and avoiding the chemical oxidation of products [39]. In this context, it is relevant to analyze whether PHA can form stable compounds with phloretin, as well as the effects this interaction has on their electron transfer capacity. To this end, we optimized the structure of some PHA interacting with phloretin. We selected some of the PHA as an example, since similar reactivity can be expected of all of them, regardless of the size of the system. We investigated three systems, two monomers (PHA-methyl and PHA-ethyl) and one trimer [(PHA-ethyl)_3_]. The results are reported in Fig. 6. The interaction energies, calculated according to Eq. (3), are also reported in this figure. As can be seen, there are not strong interactions. PHA and phloretin are close to each other, but the bond distances are long. The interaction energies are small. The distance between phloretin and the PHA is small, but no new bonds are broken or formed in either molecule. With these interaction energy values, it can be stated that there are weak non-covalent interactions. PHA functionalized with phloretin do not lead to the formation of new compounds. The interaction energies are close to 10 kcal/mol and indicate that there are not strong bonds formed between the PHA and the phloretin, with the interaction taking place through hydrogen bonds. As a result of these weak non-covalent interactions, the electron donor acceptor properties of these systems are different.

**Fig. 7:**
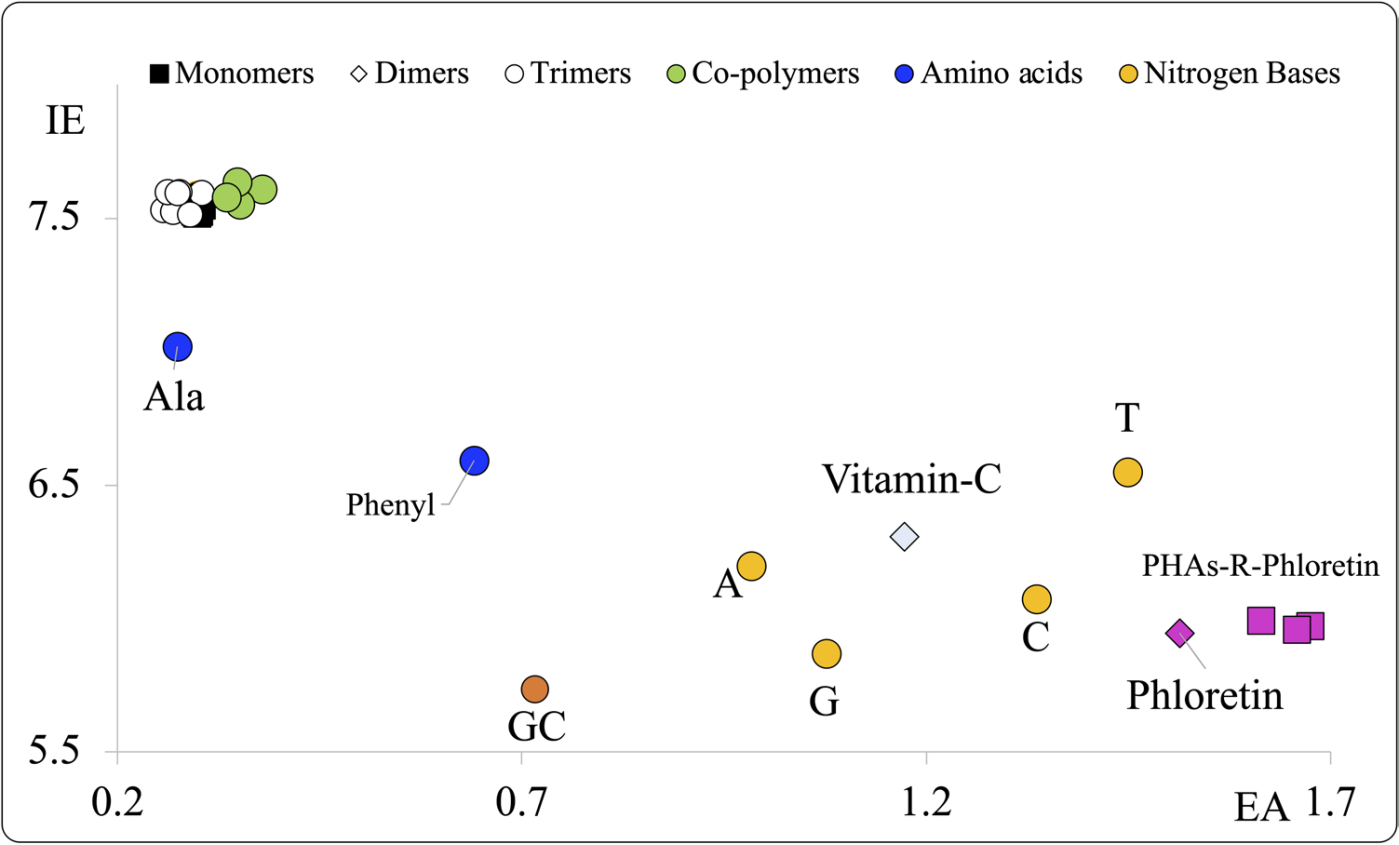
FEDAM of the most stable optimized structures of the systems studied in this investigation. In this Fig. PHA functionalized with phloretin/PHA-R-Phloretin) are included. There are other molecules such as vitamin C, phloretin. guanine-cytosine base pair (GC), adenine A), guanine (G), cytosine (C), thymine (T), alanine (Ala) and phenylalanine (Phenyl). All values are in eV. IE and EA are calculated with Eqs. (1) and (2)

To analyze the electron transfer capacity of phloretin interacting with PHA, we present the FEDAM including these new systems in Fig. 7. Phloretin is a good electron acceptor, with large EA value. Figure 7 indicates that the electron transfer capacity of PHA is improved in the presence of phloretin, even though no bond formation or dissociation occurs between PHA and phloretin. The electron transfer properties of PHA change, where they become good electron acceptors and also good electron donors with the presence of phloretin. This explains the effectiveness of functionalized PHA, which allow us to create packaging materials capable of acting against pathogenic bacteria and, therefore, preventing food spoilage. This shows that the weak interaction with phloretin makes them active to the electron transfer process. This is also in agreement with the idea that PHA functionalized with phloretin are materials with antioxidant properties [39].

## Conclusions

All the oligomers investigated exhibit the same electron donation and acceptance properties, regardless of the system size. This differs from what was observed previously with other oligomers, whose ability to accept and donate electrons was shown to depend on the size of the system. For PHA (polymers and copolymers) size doesn’t matter. They are all the worst electron donors (highest EI values) and the worst electron acceptors (lowest EA values). The results reported here indicate that these nanoplastics are not able to transfer electrons. Therefore, it is not obvious that they can contribute to oxidative stress in animals and plants. The electron transfer capacity of PHA is improved in the presence of phloretin, even though no bond formation or dissociation appears between PHA and phloretin. The electron transfer properties of PHA change, where they become good electron acceptors and also good electron donors with the presence of phloretin. This explains the effectiveness of functionalized PHA, which allow to create packaging materials capable to act against pathogenic bacteria, preventing food spoilage.

The results presented here provide information on the potential toxicity of these nanoplastics. It is possible to state with certainty that these oligomers do not participate in oxidative stress, at least not through the electron transfer mechanism. Nanoplastics are present everywhere, but the damage they cause is not evident. New studies considering realistic concentrations, dose-dependent effects, and individual susceptibility are required, but this is beyond the scope of this investigation. For the moment it is important to know that oligomers of PHA do not contribute to the oxidative stress. This will be useful for further studies.

### Future perspectives

Reports on PHA have grown exponentially. PHA-derived nanoplastics are known to be less harmful to the environment than non-biodegradable polymers such as polyethylene, PET, and synthetic resins. Furthermore, PHA degradation products have been reported to be beneficial, as their presence improves gastrointestinal function, blood–brain barrier permeability, and neuroprotective properties, among other functions [56–60]. PHA-based medical materials, such as porous poly(3-hydroxyoctanoate) patches combined with diclofenac [57], poly(3-hydroxyoctanoate)-based materials [59], and bio ceramic/polyhydroxyalkanoate composites with biomedical applications, have shown potential uses in bone tissue engineering, cartilage repair, and drug delivery systems [60]. All of these new findings and PHA-related materials offer promising avenues for future theoretical research. It is also important to investigate in detail with quantum chemistry the degradation process of PHA as it was reported for other biodegradable plastics [61]. Since oligomers formed during degradation include longer chains, it is interesting for future perspective to extend calculations to oligomers containing more monomeric units. We hope that these theoretical results motivate experimental researchers to carry out experiments with spectroscopic techniques that can confirm the presence of new intermolecular interactions of great interest for the analysis of PHA biodegradability.

## Data Availability

The datasets generated during and/or analyzed during the current study are available from the corresponding author on reasonable request.
